# Non-Lethal Concentrations of CdCl_2_ Cause Marked Alternations in Cellular Stress Responses within Exposed Sertoli Cell Line

**DOI:** 10.3390/toxics11020167

**Published:** 2023-02-09

**Authors:** Yonghong Man, Yunhao Liu, Chuanzhen Xiong, Yang Zhang, Ling Zhang

**Affiliations:** 1 Department of Occupational and Environmental Medicine, School of Public Health, Wuhan University of Science and Technology, Wuhan 430060, China; 2 Center of Scientific Research and Experiment, Nanyang Medical College, Nanyang 473006, China

**Keywords:** cadmium, CdCl_2_, Sertoli cell, TM4, cellular stress responses

## Abstract

Cadmium is a component of ambient metal pollution, which is linked to diverse health issues globally, including male reproductive impairment. Assessments of the acute effects of cadmium on male reproduction systems, such as testes, tend to be based on frank adverse effects, with particular molecular pathways also involved. The relationship between cytotoxicity potential and cellular stress response has been suggested to be one of the many possible drivers of the acute effects of cadmium, but the link remains uncertain. In consequence, there is still much to be learned about the cellular stress response induced by a non-lethal concentration of cadmium in male reproductive cells. The present study used temporal assays to evaluate cellular stress response upon exposure to non-lethal concentrations of Cadmium chloride (CdCl_2_) in the Sertoli cell line (TM4). The data showed alternations in the expression of genes intimated involved in various cellular stress responses, including endoplasmic reticulum (ER) stress, endoplasmic unfolded protein stress (UPRmt), endoplasmic dynamics, Nrf2-related antioxidative response, autophagy, and metallothionein (MT) expression. Furthermore, these cellular responses interacted and were tightly related to oxidative stress. Thus, the non-lethal concentration of cadmium perturbed the homeostasis of the Sertoli cell line by inducing pleiotropic cellular stresses.

## 1. Introduction

Cadmium (Cd) is one of the major heavy metals in environmental pollutants [1]. During natural and anthropic processes, Cd and its compounds, such as Cd-chloride (CdCl_2_), are produced [2]. These chemicals enter the food chain by spreading into the soil, water, and air, where the general population is subjected to environmental and professional exposure [3]. Among the most environmentally exposed population are tobacco smokers, who are estimated to absorb about 1–3 μg Cd from smoking one pack of cigarettes per day, resulting in double Cd body charge compared to nonsmokers [4]. Such an inhalation of Cd also occurs exclusively in professional exposure [5]. In contrast to the definite exposure route, the relationship between endogenous Cd concentration and its adverse effects in humans, including testicular damage, remains controversial. Epidemiological observational studies on populations with both environmental exposure and occupational exposure have suggested inconsistent findings [6,7]. The inconsistency, concerning the correlation between local Cd concentration and male reproductive function, has not been fully addressed.

Experimental studies, in vivo and in vitro, have shown adverse effects of Cd toxicity, which include a deleterious influence on Sertoli cells [8,9]. These somatic cells of the testis are epithelial supporting cells in seminiferous tubules. Deriving from epithelial sex cords of the gonads from embryonic stages, Sertoli cells play a pivotal role in testis development and spermatogenesis [10,11]. The interplay between Sertoli cells and surrounding somatic counterparts, as well as germline cells, is linked to the overall testicular function and productional disorders, including azoospermia and infertility [12,13]. However, the Sertoli cell is very susceptible to Cd exposure. Cd^2+^ ions or Cd^2+^ complexed with small organic molecules are transported across cellular membranes via ion channels, carriers, and ATP hydrolyzing pumps, whereas receptor-mediated endocytosis (RME) internalizes Cd^2+^-protein complexes [14]. The accumulation of Cd in cells has been linked to various cellular stress responses, which include endoplasmic reticulum stress, autophagy [15,16], mitochondrial unfolded protein stress (UPRmt) [17], mitochondrial dynamics [18,19], and Nrf2-related antioxidative response [20]. In addition, Metallothioneins (MTs) are also known to be involved in cellular defense mechanisms against heavy metal exposure [21,22]. As the cytotoxicity of chemicals is derived from the interaction between specific insults and consequent cellular stress responses [23], research studies, focusing on protective activities of cellular stress responses, are also required to fully understand the cytotoxicity of Cd, which has not been explored systematically in Sertoli cells. Moreover, although previous studies have shown adverse effects of cadmium on Sertoli cells, the mechanism underlying the cytotoxicity, especially from a non-lethal concentration of cadmium, remains uncertain at present.

To investigate the cytotoxicity of cadmium more globally, cellular stress responses, which have been suggested to be associated with protective activities upon heavy metal perturbance, were studied temporally after treatment with non-lethal concentrations of CdCl_2_ in the Sertoli cell line. These data showed that CdCl_2_ exerted a temporal influence on cellular stress responses. Remarkably, the biochemical analysis suggested an essential role of CHOP, p62, HO1, and MT in response to cadmium stimulus in Sertoli cells. These observations provide biological data for further investigation into the correlation between local Cd concentration and male reproductive function under pathophysiological conditions.

## 2. Materials and Methods

### 2.1. Cell Culture

The mouse Sertoli cell line TM4 (ATCC number: CRL-1715) was purchased from the Procell Life Science&Technology Co., Ltd., (Wuhan, China). TM4 was grown at 37 °C in a 5% CO_2_ atmosphere in DMEM/F12 (1:1) Medium, supplemented with 5% fetal bovine serum and 1% antibiotic cocktail (penicillin/streptomycin). When cells reached 80% confluence, they were subjected to incubation under the conditions described in the next section.

### 2.2. Experimental Conditions

Cadmium chloride (CdCl_2_) was purchased from Sigma-Aldrich (St. Louis, MO, USA), and its stock solution was prepared in phosphate-buffered saline (PBS) at a concentration of 100 mM. The stock solution was divided into small sterile tubes (20 μL) and stored at −20 °C before cell treatment. TM4 (density 3 × 10^4^/cm^2^) was seeded in plates with DMEM/F12 (1:1) supplemented with a low concentration of FBS (1%). After culture for 12 h, they are treated with different conditions as followed: (a) culture medium alone; (b) 1 μM CdCl_2_ and incubation medium; (c) 5 μM CdCl_2_ and incubation medium; and/or (d) 10 μM CdCl_2_ and incubation medium.

### 2.3. Formazan Formation Assay

To determine the cell viability, a sensitive colorimetric assay was performed using Cell Counting Kit-8 (Dalian Meilun Biotechnology Co., Dalian, China) according to the manufacturer’s instructions. The optical densities (OD) of the water-soluble formazan dye were measured at 450 nm using a microplate reader (EPOCH2, BioTek, Winooski, VT, USA).

### 2.4. Cellular ROS Assay

At the end of the reattachment period (12 h), the incubation medium was replaced with a serum-free medium containing 10 μM DCFH-DA (Beyotime Institute of Biotechnology, Haimen, China). Then, the cells were incubated at 37 °C for 20 min. After triple washes with serum-free medium to remove free DCFH-DA completely, the cells were subjected to incubation under indicated experimental conditions for 2 h. The fluorescence intensity was monitored at 480 nm with a ZOE Fluorescent Cell Imaging System (Bio-rad, Hercules, CA, USA).

### 2.5. TUNEL Assay

At the end of the incubation period (12 h), the cells were washed with PBS followed by a fixation with 4% PFA for 20 min and permeabilization with 0.3% Triton X-100 (PBS) at room temperature for 5 min. Then, the cells were incubated with a combination of TdT enzyme and fluorescent labeling solution (Beyotime Institute of Biotechnology, Haimen, China) at 37 °C for 60 min. After triple washes with PBS, the fluorescence intensity was monitored with the ZOE Fluorescent Cell Imaging System (Bio-rad, Hercules, CA, USA) at 480 nm.

### 2.6. RNA Extraction, cDNA Synthesis

At the end of the incubation, total RNA was extracted with Trizol reagent (Kangwei, Ltd., Guangzhou, China) according to the manufacturer’s instructions. After removal of the culture medium, Trizol (1 mL/well) was added to the culture well. For adequate lysis of cells, the plate was shaken on a circumferential shaker (Dalong, Beijing, China) with a speed of 100 rpm/min for 10 min at room temperature. The lysate was transferred into RNase-free microtubes, and 200 μL chloroform was added to each microtube. After a violent vortex, the microtube was centrifugated using a Fresco 17 centrifuge (Thermo Fisher Scientific, Waltham, MA, USA). Next, about 420 μL supernatant (Aquos phase) was acquired and transferred into a new microtubule, and an equal volume of 70% ethanol was added and mixed gently. The mixture was loaded into Spin Columns for RNA purification. Finally, the total RNA was eluted with 50 μL RNase-free water, and, thereafter, the quality and amount of RNA samples were determined with a spectrophotometer (Thermo Fisher Scientific, USA). Acceptable RNA samples have a ratio of 260/280 nm (1.8–2.0). To synthesize cDNA, PrimeScript™ RT reagent Kit (Takara, Japan) was used. DNA contamination was cleared using DNase according to the manufacturer’s protocol, and a 20 μL reaction mixture of reverse transcription, containing 250 ng template RNA, 4 μL PrimeScript Buffer (5×), 1 μL Enzyme mix, and 1 μL RT Primer Mix, was used based on the manufacturer’s protocol. The cDNA cycling protocol includes two steps: 37 °C for 15 min followed by 85 °C for 5 s.

### 2.7. Quantitative Polymerase Chain Reaction (qPCR)

A 2× Real-Time PCR master mix (Takara, Japan) was used to perform the qPCR assay. A standard reaction mixture was prepared according to the manufacturer’s instructions. Furthermore, the protocol of qPCR was performed with the default parameters of the 7500 Fast Real-Time PCR System (Applied bio. Waltham, MA, USA). To check the specificity of amplification, a melt curve was used after the amplification cycles had been completed. The primer sequences used for qPCR are shown in Table S1. The reactions were performed in triplicate or quadruplicate. A reference PCR involving the endogenous housekeeping gene GAPDH was used to normalize the mRNA level of each gene, and levels of gene expression were determined with the comparative CT method (2^−ΔΔCT^ method).

### 2.8. Protein Preparation

Cellular extracts were prepared using a cell lysis buffer (20 mM Tris, 150 mM NaCl, 1% Triton X-100 and inhibitor cocktails), supplemented with 1 mM Phenylmethanesulfonylfluoride (PMSF). The cellular lysate was then centrifuged with a speed of 12,000× *g* at 4 °C for 5 min, and the supernatant was collected. The protein concentration of the samples was determined using BCA assay, and the OD values were measured at 562 nm using a microplate reader (EPOCH2, BioTek, Winooski, VT, USA). The protein samples prepared were either analyzed immediately or stored at −80 °C for future analysis.

### 2.9. Immunoblotting Analysis

The protein samples, mixed with a 6× loading buffer (Beyotime Institute of Biotechnology, Haimen, China), were heated at 95 °C for 5 min. Equal amounts of protein were separated by SDS-PAGE and transferred to polyvinylidene fluoride membranes (Immobilon-P, Millipore, Bedford, MA, USA). Membranes were blocked in Phosphate-buffered saline (PBS) plus 0.1% Tween 20 (PBST) and 5% nonfat dry milk. Membranes were then incubated with primary antibody diluted in 5% milk-PBST at 4 °C overnight. Membranes were subsequently washed in PBST and incubated with horseradish peroxidase (HRP)-labelled antibodies (Proteintech, USA). The protein bands were detected with enhanced chemiluminescence assay. The intensity of protein bands was analyzed with ImageJ software. Antibodies used for this study are listed in Table 1.

### 2.10. MDA Assay

MDA levels of protein samples from each group were measured by the colorimetric Thiobarbituric Acid (TBA) method. In brief, the protein sample was mixed with TBA and heated at 100 °C for 15 min. After being cooled to room temperature, the mixture was centrifuged at 1000 g for 10 min, and the colorimetric absorption of the supernatant was measured at 535 nm.

### 2.11. Dual-Labeled Immunofluorescence Analysis

TM4 cells cultured on coverslips were fixed in 4% paraformaldehyde in PBS (10 mM sodium phosphate and 0.15 M NaCl, pH 7.4, at 22 °C; *w/v*) for 10 min and permeabilized in Immunostaining Permeabilization Solution with Triton X-100 (Beyotime Ltd., Zhejiang, China) for 5 min. Cells were then blocked with 5% goad serum in PBS (*v/v*; blocking solution), followed by overnight incubation at 4 °C with primary antibodies diluted in blocking solution: mouse anti-p62 (Proteintech; 1:100). Thereafter, cells were washed in PBS and incubated with secondary fluorescent antibody Alexa Fluor–conjugated secondary antibodies (Abcam, Cambridge, UK; red fluorescence, Alexa Fluor 555) at 1:250 dilution with blocking solution at room temperature for 1h. For mitochodrion staining, cells were only incubated with Mito-Tracker Red CMXRos (Beyotime, 1:1000) diluted in culture medium. Cells were then washed and mounted with Antifade Mounting Medium with 4-,6-diamidino-2-phenylindole (DAPI; beyotime). Fluorescence images were captured using Eclipse Ci-L Fluorescence Microscope (Nikon, Tokyo, Japan).

### 2.12. Statistical Analysis

All experiments were successively repeated three or four times. Reliable data meet the condition SD/mean <10%. The heat map was expressed as a mean value (*n* = 3). All data were analyzed by the independent, two-tailed Student’s t-test. The data are presented as mean ± SD (standard deviation). Probability values less than 0.05 were regarded as significant.

## 3. Results

### 3.1. Subsection

#### 3.1.1. The Viability of TM4 Cells upon CdCl_2_ Treatment

The non-lethal concentrations of CdCl_2_ were determined based on the following experiments. TM4 cells were treated with different concentrations of CdCl_2_ for 1, 3, 6, 12, and 24 h, respectively.

**Figure 1:**
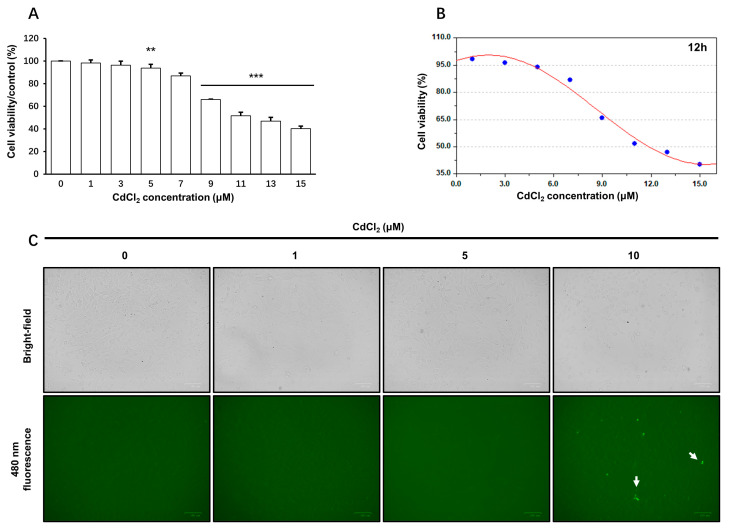
CdCl_2_ treatment reduced cell viability and induced apoptosis in a dose-dependent manner. The cell viability was evaluated by using the CCK8 test in TM4 cells after incubation for 12 h, in the presence of CdCl_2_. Data were significantly different between CdCl_2_ (5~15 μM) treatment and controls as determined by using the *t*-test (*p* < 0.01) (**A**). The inhibition effects of CdCl_2_ were also estimated using the Sine Fitting algorithm, where an IC_50_ was deduced as 11.8 μM at 12h (**B**). The cellular apoptosis was detected with a TUNEL assay. Representative images illustrate the presence of TdT-mediated dUTP Nick-End Labeling (white arrow) showing DNA breaks during apoptosis (**C**). ** *p* < 0.01 compared to the control group, and *** *p* < 0.001 compared to the control group.

CCK-8 assay was used to determine the production of soluble formazan. Cells exposed to 5 μM~15 μM CdCl_2_ showed a significant decrease in formazan production after 12 h exposure (approximately 6~60% inhibition of control values; *p* < 0.05) (Figure 1A). This inhibition was increased to approximately 24~89% (*p* < 0.05) after an incubation period of 24 h (Figure S1). Therefore, a lower concentration (<5 μM) of CdCl_2_ did not affect WST-8 reduction at any indicated time point, whereas 5 μM was the lowest concentration to affect the formazan formation ability of the Sertoli cell line at both 12 and 24 h. The toxic effects of CdCl_2_ were further compared in terms of IC50 value, which was estimated to be 11.8 and 6.5 μM at 12 and 24 h (Figure 1B), respectively. Bright-field observation also showed no remarkable morphological alternations after treatment with ≤5 μM CdCl_2_ for 12 h (Figure S2). Furthermore, the apoptosis of cells was monitored with a One Step TUNEL Apoptosis Assay Kit (Beyotime, Shanghai, China). This assay detects apoptotic cells by labeling blunt ends of double-stranded DNA breaks during the late stages of apoptosis. Furthermore, treatment with CdCl_2_ (1 and 5 μM) did not increase the fluorescent labeling (bright green dots) compared with the control group (Figure 1C). Thus, treatments with 1 and 5 μM CdCl_2_ were not linked to significant cellular death within the indicated time and were used in the low-concentration group and high-concentration group, respectively. For further details concerning WST-8 reduction assay, see Figure S1.

#### 3.1.2. Effects of CdCl_2_ Treatment on Endoplasmic Reticulum Stress

**Figure 2:**
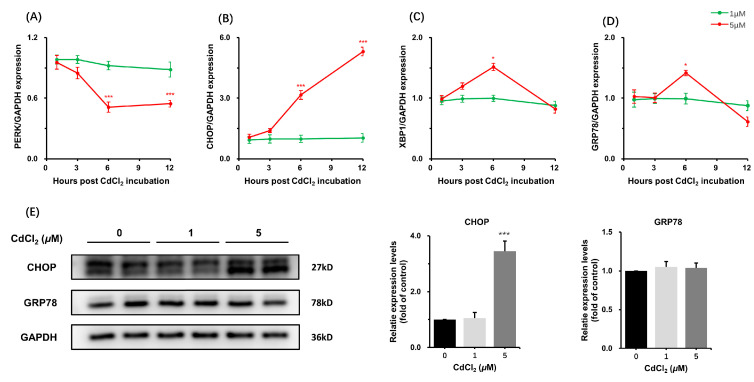
CdCl_2_ treatment induced the endoplasmic reticulum stress in the Sertoli cell. The mRNA levels of (**A**) PERK, (**B**) CHOP, (**C**) XBP1, and (**D**) GPR78 were measured by using qPCR at 1, 3, 6, and 12 h after treatment with CdCl_2_ (1, 5 μM). The protein levels of CHOP and GRP78 at 12 h were evaluated by immunoblotting (**E**). * *p* < 0.05 compared to the control group, *** *p* < 0.001 compared to the control group.

Endoplasmic Reticulum (ER) stress has been implicated in Cd-induced cytotoxicity across various cell types. This Cd-induced cell response, however, is barely investigated in the Sertoli cell. Given that PERK, HSPAS, CHOP, and GRP78 have been widely used for monitoring ER stress, we investigated the dynamic expression of these marker genes. Treatment with 5 μM CdCl_2_, but not 1 μM, significantly altered the mRNA levels during the early stage of the treatment period (3–12 h). In addition, the mRNA level of CHOP in the 5 μM group showed a >5-fold increase compared to other groups (Figure 2A–D). Western-blot analysis showed a concomitant increase in CHOP protein level at 12 h (Figure 2E). These data showed a significant effect of CdCl_2_ on ER stress in both time- and dose-dependent manners.

#### 3.1.3. Effects of CdCl_2_ Treatment on Mitochondrial Unfolded Protein Response (UPRmt) and Mitochondrial Dynamics

**Figure 3:**
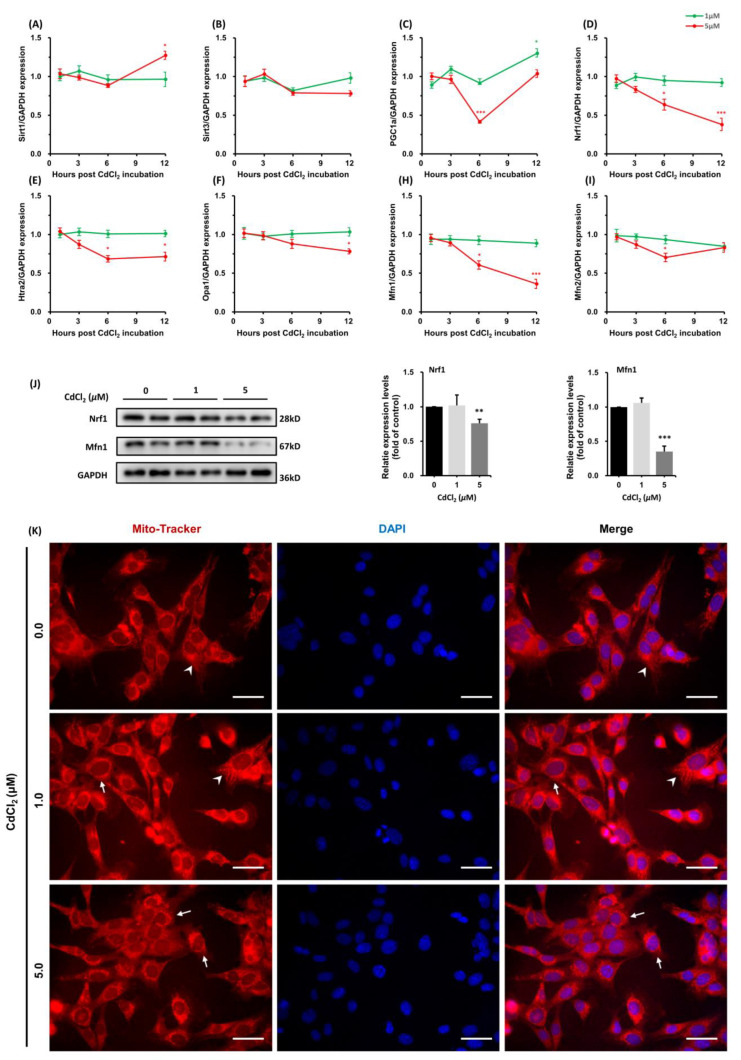
CdCl_2_ disturbed mitochondrial homeostasis in the Sertoli cell line. The mRNA levels of genes related to UPRmt (**A**–**E**) and mitochondrial dynamics (**F**–**H**) were measured by using qPCR at 1, 3, 6, and 12 h after treatment with CdCl_2_ (1, 5 μM). The protein levels of Nrf1 and Mfn1 at 12h were evaluated by immunoblotting (**I**). Note that CdCl_2_ treatment induced an alternation in mitochondria morphology as show with Mito-Tracker staining (**J**). The tubular mitochondria is marked with an arrowhead, and the dotted mitochondria is marked with an arrow. Scale bar 50 μm for immunofluorescence images. * *p* < 0.05 compared to the control group, ** *p* < 0.01 compared to the control group, and *** *p* < 0.001 compared to the control group.

Cadmium was recently shown to interfere with the mitochondrial unfolded response (UPRmt), and it disrupted the homeostasis of mitochondrial dynamics in the kidney. In consistency with CdCl_2_-induced changes in the kidney, most genes related to UPRmt and mitochondrial dynamics were downregulated in 5 μM groups (Figure 3A–H). Among the genes, that the expression was reduced significantly, in ***Nrf1*** and ***Mfn1***. Additionally, the expression fold was reduced to 0.38 ± 0.02 (*p* < 0.01) and 0.36 ± 0.02 (*p* < 0.01), respectively. Immunoblotting also showed a concomitant decrease in Nrf1 and Mfn1 protein levels (Figure 3I). Moreover, CdCl_2_ treatment also led to changes in mitochondrial morphology. Tubular or filamentous mitochondria were regarded as normal whereas dotted mitochondria were deemed to be damaged. The treatment with CdCl_2_ induced a significant increase in dotted mitochondria compared with blank control (Figure 3J). These alternations suggested that a higher concentration of CdCl_2_ caused a disturbance in the homeostasis of mitochondrial activity.

#### 3.1.4. Effects of CdCl_2_ Treatment on Nrf2 Antioxidative Response-Related Genes

**Figure 4:**
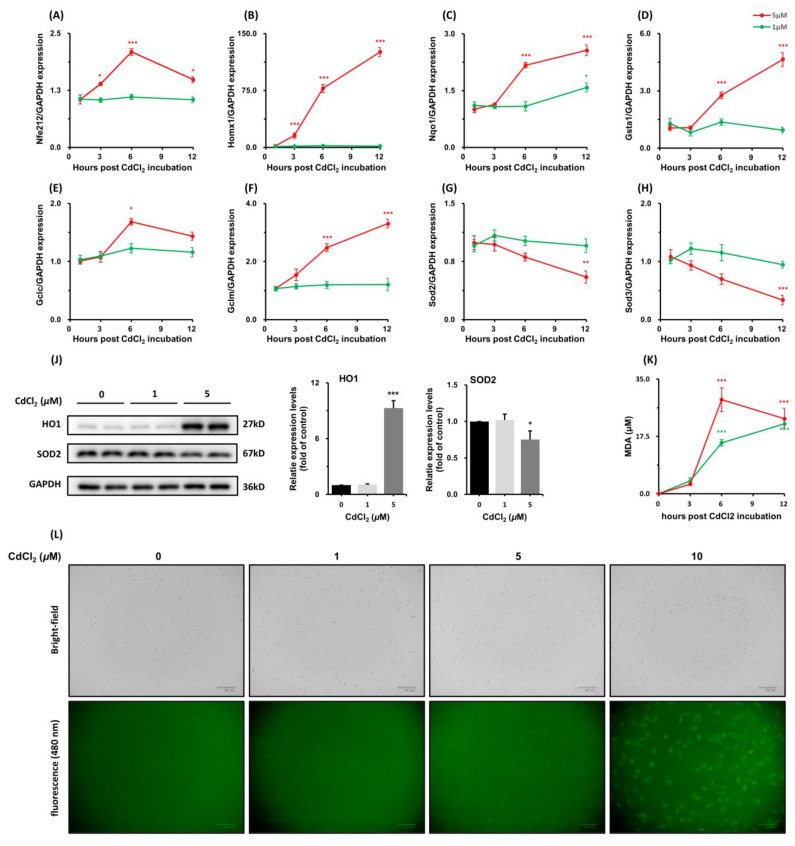
CdCl_2_ induced oxidative stress in TM4. The mRNA levels of Nrf2-related genes were measured using qPCR after treatment with CdCl_2_ for 1, 3, 6, and 12 h (**A**–**H**). The protein levels of HO1 and SOD2 at 12 h were measured by immunoblotting (**I**). ROS level induced by treatment with CdCl_2_ for 2 h was measured by a fluorescence probe (DCFH-DA). Representative images illustrate the presence of fluorescent DCF (**K**), an oxidation product of DCFH oxidized by ROS. The MDA levels were measured after treatment with CdCl_2_ for 1, 3, 6, and 12 h (**J**). * *p*< 0.05 compared to the control group, ** *p* < 0.01 compared to the control group, and *** *p* < 0.001 compared to the control group.

Cadmium can increase the production of ROS and resultant oxidative stress, leading to enhanced antioxidative activity. CdCl_2_, especially in high concentrations, causes an upregulation of genes involved in the Nrf2 antioxidative response. The most induced antioxidative enzyme was HO1 (Figure 4B). An amount of 5 μM CdCl_2_ induced a >140-fold increase in mRNA level of HO1 after 12 h. The transcriptional increase was consistent with its protein levels (Figure 4J). The expression of Sod2 and Sod3 was reduced across the time course (Figure 4H,I). In contrast, CdCl_2_ induced an increase in ROS production in a dose-dependent manner at the early stage (Figure 4K), and Malondialdehyde (MDA) concentration was also increased at 6 h significantly (Figure 4J, *p* < 0.001). The MDA is formed via the peroxidation of polyunsaturated fatty acids and is a significant biomarker of oxidative stress. These data are reflective of ROS insults and enhanced antioxidative activities in the Sertoli cell line in response to CdCl_2_ treatment.

#### 3.1.5. CdCl_2_ Caused Interferences in the Expression of Autophagy-Related Genes and Increases in Metallothioneins (MTs) Transcription

**Figure 5:**
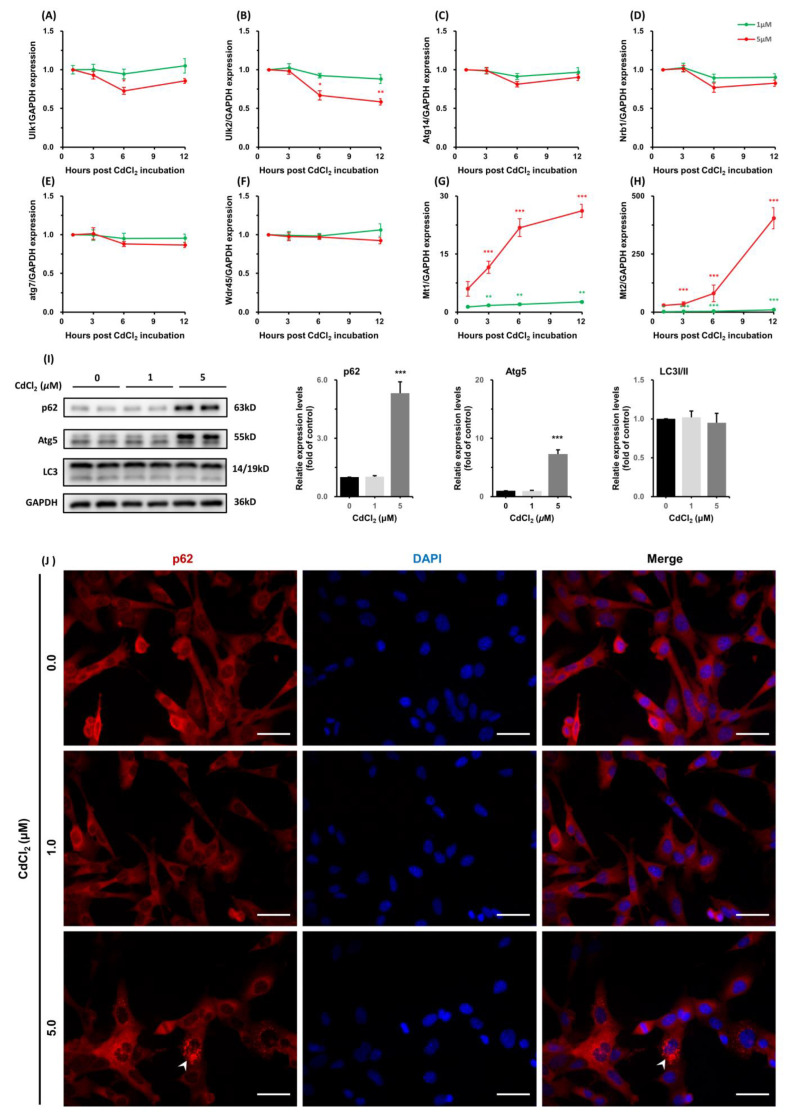
CdCl_2_ suppressed the autophagy activities and increased MT transcription in TM4. The mRNA levels of genes monitoring autophagy were measured using qPCR after treatment with CdCl_2_ (1, 5 μM) for 1, 3, 6, and 12 h (**A**–**F**). The protein levels of p62, Atg5, and LC3 after treatment with CdCl_2_ (1, 5 μM) for 12 h were measured by immunoblotting (**I**). Changes in expression of cellular p62 (red) were examined by immunofluorescence microscopy (**J**). The mRNA levels of MT1 and MT2 were also measured at the indicated time (**G**,**H**). Scale bar 50 μm for immunofluorescence images. *** *p* < 0.001 compared to the control group.

Autophagy is a lysosomal degradation pathway that regulates cellular homeostasis. To determine the effects of CdCl_2_ on autophagy, we measure the expression of a list of genes, which has been established as a gene toolbox for monitoring autophagy. These genes, including ***Atg14***, ***Atg7***, ***Nbr1***, ***Ulk1***, ***Ulk2***, and ***Wdr45***, showed suppression of transcription across the incubation period (Figure 5A–F). Furthermore, CdCl_2_ (5 μM) induced an increase in the protein levels of p62 and Atg5 at 12 h, without a significant alternation in the ratio of LC3I to LC3II (Figure 5I). The expression of p62 was also confirmed using cellular immunofluorescence. CdCl_2_ treatment increased the intensity of p62 fluorescence signals (bright red) in the cytoplasm (Figure 5J).

Metallothioneins (MTs) are small cysteine-rich proteins that play important roles in metal homeostasis and protection against heavy metal toxicity, DNA damage, and oxidative stress. CdCl_2_ induced a potent and acute increase in transcripts of both ***Mt1*** and ***Mt2*,** which are the dominant MTs in Sertoli cells. The mRNA levels of MT1 and MT2 were upregulated >20 fold (Figure 5G) and >400 fold (Figure 5H), respectively, after treatment with 5 μM CdCl_2_ for 12 h.

**Figure 6:**
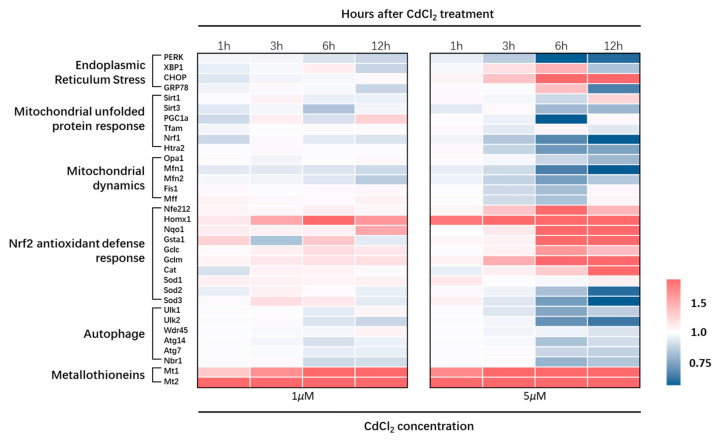
The heat map indicates the dynamic gene expression of cellular response after treatment with CdCl_2_ in TM4.

The overall transcriptional dynamics of genes involved in the cellular response to CdCl_2_ treatment are shown in Figure 6. And transcriptional change of *Sod1*, *Mff*, and *Tram* are listed in Figure S3.

## 4. Discussion

This study provides several new insights into the molecular response to cadmium exposure in Sertoli cells. CdCl_2_ in non-lethal concentrations induced Nrf2 antioxidative response and ER stress, alongside interferences with mitochondrial unfolded protein response (UPRmt), mitochondrial dynamics, and autophagy. Moreover, CdCl_2_ induced a potent and sustained increase in metallothionein (MT) expression, establishing a possible particular role in cellular resistance to cadmium cytotoxicity in the Sertoli cell.

Endoplasmic reticulum (ER) stress is a basic cellular stress response that maintains cellular protein homeostasis under endogenous or exogenous stimuli [24]. Cd has shown the potential to induce ER stress in various cell types, which has been implicated in neurotoxicity, nephrotoxicity, and hepatotoxicity [16,25,26]. Cd triggers the PERK-eIF2a-ATF4 pathway, induces splicing of XBP1 mRNA, and increases CHOP expression [17]. The present study confirms and expands upon those findings by showing that CdCl_2_ also induced ER stress in the Sertoli cell line. As CHOP plays an important role in ER stress-induced apoptosis [27], the increased CHOP also suggests that CdCl_2_ might cause apoptosis via PERK/ATF4/CHOP Signaling Pathway in the Sertoli cell. Moreover, overexpression of CHOP can lead to cell cycle arrest by inhibiting the expression of cell cycle regulatory protein, p21 [28]. This relationship might provide a possible explanation for reduced Forzan formation in the 5 μM CdCl_2_ group.

Mitochondria dynamics are characterized by coordinated cycles of fission and fusion of mitochondria. The interplay of fusion and fission confers widespread benefits on mitochondria, including efficient transport, increased homogenization of the mitochondrial population, and efficient oxidative phosphorylation [29]. A beneficial mitochondrial dynamic depends on balanced fusion, which is orchestrated by Opa1, Mfn1, and Mfn2, and fission by Fis1 and Mff [30]. Cd has shown great influence on mitochondria dynamics in cortical neurons [18,19]. However, these influences can be context- and stage-dependent. In the present study, CdCl_2_ suppressed the expression of Opa1 and Mfn1, both of which mediate mitochondrial fusion. Reduced mitochondrial fusion contributes to the segregation of damaged mitochondria for degradation and release of pro-apoptotic factors [16]. This alternation may be reflective of cellular response to mitochondria impairment, but also might suggest an apoptosis pathway triggered by a higher concentration of CdCl_2_ in Sertoli cells. To support this notion, Cd has recently been shown to selectively trigger oxidative stress and mitochondrial injury-mediated apoptosis [31].

Mitochondria unfold protein response (mtUPR) is a homeostatic mechanism. Upon mitochondrial dysfunctions, mtUPR contributes to the restoration of mitochondrial proteostasis and hence biogenesis [32]. The canonical mtURP includes three axes of the mtUPR program: SIRT3-mtUPR, ATF5-mtUPR, and ERa-mtUPR. These biological processes have shown interactions with various signal pathways [33]. Here, we presented evidence for CdCl_2_-induced interference with mtURP in the Sertoli cell line. The reduced expression of mt-UPR-related genes, including PCG1a, Nrf1, and Htra2, was also observed in the *Gallus gallus* model of cadmium exposure [34]. It is worth mentioning that CHOP has also been implicated in the regulation of mtURP within the mammalian system [32]. The precise nature of the interactions between mt-UPR and CHOP warrants further investigation in Sertoli cells upon Cd exposure.

Excessive ROS production in tissues or cells can induce oxidative stress. Multiple cellular processes, such as dysregulated mitochondria and ER stress, contribute to ROS production [35]. Furthermore, oxidative stress might be one of the major mechanisms responsible for Sertoli cell damage by Cd, because ROS were increased in TM4 after CdCl_2_ treatment for 2 h, along with altered expression of genes related to mitochondrial activities and ER stress.

Nrf2 plays a key role in regulating cellular resistance to oxidants [36]. During oxidative stress, Nrf2 is released either through its dissociation from Kelch-like ECH-associated protein 1 (Keap1) or by the upregulation of SQSTM/p62 [37]. Nrf2 mediates the antioxidative response by regulating the expression of a group of antioxidative genes, which share a common DNA sequence called the antioxidant response element (ARE) [36]. Cd exposure has been linked to an increase in the expression of ARE-containing genes [38]. The present study also identified substantial activities of Nrf2 antioxidative response, evidenced by the upregulation of antioxidative genes. However, treatment with 5 μM CdCl_2_ suppressed transcriptional levels of CAT, SOD, and NQO1 in Granulosa cells [39]. This discrepancy might suggest a context- and dose-dependent cytotoxicity of Cd. Increases in HO1 levels by CdCl_2_ incubation have been previously reported [40]. Our data further contextualizes this increase as one of the most notable transcriptional changes in Nrf2-regulated antioxidant defense. Heme Oxygenase 1 (Homx1), which is encoded by HO1, catalyzes the rate-limiting step in heme degradation, producing equimolar amounts of CO, biliverdin, and iron ions [41]. The accumulated iron can drive a generation of lipid ROS and resultant cell death, namely ferroptosis. As a consequence, dysregulation of Homx1 might also contribute to the cytotoxicity of Cd via ferroptosis in the Sertoli cell. To support this notion, Cd has been shown to induce ferroptosis in various models, both in vitro and in vivo [15]. Moreover, Cd exposure enhanced the binding of Nrf2 to ARE promoter regions of p62/Bcl-2 [42], and the increased expression of p62 was also observed in the present study. Thus, the cytotoxicity of cadmium in the Sertoli cell might be tightly associated with Nrf2/p62 signaling.

Cadmium has been shown to affect autophagy flux in various studies. CdCl_2_ (5 mg/kg/day, p.o.) exposure for 4 weeks increases the testicular protein level of p62 and activates the mTOR pathway [42], which has been known to negatively regulate autophagy. Additionally, in mouse testes, injection with CdCl_2_ (2.0 mg/kg) causes a temporal increase in the expression of autophagy-related genes including Atg5, Atg7, p62, and LC3B-II [8]. Note that LC3-II is a well-established marker for autophagy flux, and an increased LC3-II is generally related to the upregulation of autophagy flux. In contrast, protein levels of p62 and Atg5 were also increased after treatment with CdCl_2_ (5 μM) for 12 h in this study. However, the gene tools, which have been established to monitor the autophagy flux and LC3B-II showed no changes in both CdCl_2_ treatment groups across the treatment period. This discrepancy in the expression of markers for autophagy flux might be based on the toxicology characteristics of Cd. The dosage and time course of Cd treatment might exert a variable influence on the autophagy activities in the Sertoli cell line. As p62 is degraded during the autophagic process, an accumulation of p62 might also indicate a suppression in autophagy flux. This suppression has also been observed in a variety of studies focusing on Cd’s potential for carcinogenesis [43]. Moreover, the increased Atg5, paralleled with the activation of the PERK/ATF4/CHOP pathway, provided evidence for crosstalk between ER stress and autophagy during Cd exposure in Sertoli cells, which is still a process largely obscure [44].

Metallothioneins (MTs) are ubiquitous low molecular weight proteins and are characterized for binding divalent transition ions, such as Zn^2+^, Cu^2+^, and Cd^2+^ [2]. Cd exposure induces MT synthesis in the liver, kidney, and other tissues [45]. A combination treatment of Cd and Mt reduces Cd-induced tissue toxicity. This protective activity has been observed in the TM4 cell line. A pretreatment of 2 μM CdCl_2_ reduced the cytotoxicity of 100 μM Cd by increasing the expression of MT [46]. The present study confirmed these findings and showed a quick and potent increase in MT expression. Additionally, this MT response occurred in both dose- and time-dependent manners.

The present study also shows that the Sertoli cell presented with various activities in response to different concentrations of CdCl_2_. These observations demonstrate that both dosage and time course play pivotal roles in the mechanism underlying Cd cytotoxicity. Furthermore, the adverse outcomes induced by Cd exposure could be based on complicated interactions among multiple cellular processes in the Sertoli cell.

## 5. Conclusions

Taken together, the results from the present study revealed temporal cellular responses to Cd exposure in the Sertoli cell line. CdCl_2_ of non-lethal concentrations were able to perturb homeostasis of redox, mitochondrial activities, and autophagy, as well as trigger ER stress and Nrf2 antioxidant defense response. These observations support a central role of ROS in cadmium cytotoxicity within Sertoli cells. Moreover, the sensitive and marked upregulation of MT expression suggests a protective mechanism critical for metal homeostasis in Sertoli cells, and a biological marker potential in screening male productive disorders associated with Cd exposure. The framework developed here can also be used to explore mechanisms that drive the cytotoxicity of heavy metal pollutants.

## Figures and Tables

**Table 1:** Antibodies used for different experiments in this report.

Antibody	Host Species	Vendor	Catalog Number	Applications (s)/Dilutions (s)
SQSTM1/p62	Rabbit	CST	39,749	IB (1:1000), IF (1:100)
Atg5	Rabbit	CST	12,994	IB (1:1000)
Atg7	Rabbit	CST	8558	IB (1:1000)
LC3	Rabbit	ProteinTech	14,600-1-AP	IB (1:2500)
GAPDH	Mouse	ProteinTech	60,004-1-Ig	IB (1:50,000)
CHOP	Rabbit	ProteinTech	15,204-1-AP	IB (1:1000)
GRP78/BIP	Rabbit	ProteinTech	11,587	IB (1:3000)
HO-1	Rabbit	ProteinTech	10,701-1-AP	IB (1:3000)
Nrf1	Rabbit	ProteinTech	12482-1-AP	IB (1:2000)
Mfn1	Rabbit	ProteinTech	13798-1-AP	IB (1:1900)

## Data Availability

All data supporting reported results can be found in the article and supplementary materials.

## Supplementary Materials

The following supporting information can be downloaded at: https://www.mdpi.com/article/10.3390/toxics11020167/s1, Figure S1: Cell viability of TM4 after CdCl_2_ treatment; Figure S2: Morphological changes of TM4 after incubation with CdCl_2_; Figure S3: Transcriptional change of SOD1, Mff, and Tram; Table S1: The list of specific primers used for qPCR.
