# Supplementary material for: Non-Lethal Concentrations of CdCl2 Cause Marked Alternations in Cellular Stress Responses within Exposed Sertoli Cell Line
Source: Toxics. 2023 Feb 9;11(2):167. doi: 10.3390/toxics11020167 (PMC9962571; doi:10.3390/toxics11020167)

Supplementary Materials

Table S1. The list of specific primers used for qPCR

Gene	Reference Sequence	Primer	Amplicon Length
Gapdh*	NM_001289726.1	F 5`-GGAGAAACCTGCCAAGTATGA-3`<br>R 5`-TCCTCAGTGTAGCCCAAGA-3`	90
Mt2	NM_008630.2	F 5`-AAAGAGGCTTCCGACAAGTG-3`<br>R 5`-GCTAGGCTTCTACATGGTCTATTT	90
Mt1	NM_013602.3	F 5`-AGGGCTGTGTCTGCAAAG-3`<br>R 5`-CTGGGTTGGTCCGATACTATTT	100
Wdr45*	NM_001290792.1	F 5`-TAAAGACACCCGCCTTAACC-3`<br>R 5`-CCACTGAGAGTCCACGTATTG	88
Ulk1*	NM_001347394.1	F 5`-ACCAGGCAGACATTGAGAAC-3`<br>R 5`-AGCAGGTAGTCAGGCATAGA	95
Ulk2*	NM_013881.4	F 5`-TCCACCATCCCTTCCAGATA-3`<br>R 5`-CAGAGTCTTTGGACACCTGTAG	98
Nbr1*	NM_008676.3	F 5`-CAGAAGAGGATCGTTGGAGTG-3`<br>R 5`-TGGTTAGTGTCATGGGTGTATG	101
Atg7*	NM_001253717.2	F 5`-TCCTGAGAGCATCCCTCTAAT-3`<br>R 5`-GGCTCGACACAGATCATCATAG	106
Atg14	NM_172599.4	F 5`-AGTCTGCTGGGATGGTTAGA-3`<br>R 5`-GGAATGATGGAGGAAGTGAGTG	101
CHOP	NM_007837.4	F 5`-TCCTGTCCTCAGATGAAATTGG-3`<br>R 5`-GCAGGGTCAAGAGTAGTGAAG	91
GRP78*	NM_001163434.1	F 5`-GAGACTGCTGAGGCGTATTT-3`<br>R 5`-CAGCATCTTTGGTTGCTTGTC	100
Xbp1*	NM_013842.3	F 5`-TGGAAGAAGAGAACCACAAACT-3`<br>R 5`-CATTCCCAAGCGTGTTCTTAAC	104
PERK*	NM_010121.3	F 5`-GTTACACAGCCAGCGAAGAA-3`<br>R 5`-GGCCTCTGTACATCCCTAAGTA	102
SIRT1*	NM_019812.2	F 5`-GGCCTAATAGACTTGCAAAGGA-3`<br>R 5`-CTCAGCACCGTGGAATATGTAA	98
SIRT3*	NM_022433.2	F 5`-CTGGATGGACAGGACAGATAAG-3`<br>R 5`-TGAAGTCTTGCTGGACATAGG	84
PGC-1α*	NM_008904.2	F 5`-CGACAGCTATGAAGCCTATGAG-3`<br>R 5`-CTTCTGCCTCTCTCTCTGTTTG	106
TFAM	NM_009360.4	F 5`-AGGCCCAAGAAACCAGTTAG-3`<br>R 5`-AGAAAGTCCATGGCTACAGAAA	109
Nrf1*	NM_001164226.1	F 5`-AGCGATTGTACTCTGCATCTC-3`<br>R 5`-CCAGGATCATGCTCTTGTACTT	107
HTRA2*	NM_019752.3	F 5`-TGACTCCCAGCATCCTTATTG-3`<br>R 5`-GGAGCCCAGGATAACTTTATGA	95
Opa1	NM_001199177.1	F 5`-GTCAGGCTTTCTGGAACAGTAG-3`<br>R 5`-TTCGTCAACTGGTGGTCTTTAG	93

Mfn1	NM_024200.4	F 5`-GGAAGAGAGGGAAGACCAAATC-3`<br>R 5`-CGTGACCTCCTTGATCTTCTTC	97
Mfn2*	NM_001285920.1	F 5`-ATGCAGCAGGACATGATAGAC-3`<br>R 5`-GTCATAGCTGAGGGAGAAACAC	105
Fis1*	NM_025562.3	F 5`-GCCTGGTTCGAAGCAAATAC-3`<br>R 5`-AAGACATAGTCCCGCTGTTC	103
Mff*	NM_029409.2	F 5`-GCCTGGTTCGAAGCAAATAC-3`<br>R 5`-AAGACATAGTCCCGCTGTTC	103
Nfe2l2	NM_010902.5	F 5`-GGCTCAGCACCTTGTATCTT-3`<br>R 5`-CACATTGCCATCTCTGGTTTG	107
Hmox1*	NM_010442.2	F 5`-GTACACATCCAAGCCGAGAA-3`<br>R 5`-TGGTACAAGGAAGCCATCAC	98
Nqo1*	NM_008706.5	F 5`-CTCGAATCTGACCTCTATGCTATG-3`<br>R 5`-GATGACTCGGAAGGATACTGAAA	107
Gsta1*	NM_008181.3	F 5`-GACCAGAGCCATTCTCAACTAC-3`<br>R 5`-ATCAGGGCTCTCTCCTTCAT	75
Gclc*	NM_010295.2	F 5`-CCTCCTCCTCCAAACTCAGATA-3`<br>R 5`-GCAGTACCACGAATACCACATAG	106
Gclm*	NM_008129.4	F 5`-GTATCAGTGGGCACAGGTAAA-3`<br>R 5`-CGGGTCATTGTGAGTCAGTAG	127
Cat*	NM_009804.2	F 5`-GATGGTAACTGGGATCTTGTGG-3`<br>R 5`-GTGGGTTTCTCTTCTGGCTATG	100
Sod1*	NM_011434.1	F 5`-CTCAGGAGAGCATTCCATCATT-3`<br>R 5`-CTCCCAGCATTTCCAGTCTT	108
Sod2	NM_013671.3	F 5`-AGCGTGACTTTGGGTCTTT-3`<br>R 5`-AGCGACCTTGCTCCTTATTG	111
Sod3*	NM_011435.3	F 5`-GAACTTCACCAGAGGGAAAGAG-3`<br>R 5`-CAGTAGCAAGCCGTAGAACAA-3`	93

* These pairs of primer are designed to span at least one intronic sequence.

Figure S1: Cell viability of TM4 after incubation with $CdCl_2$

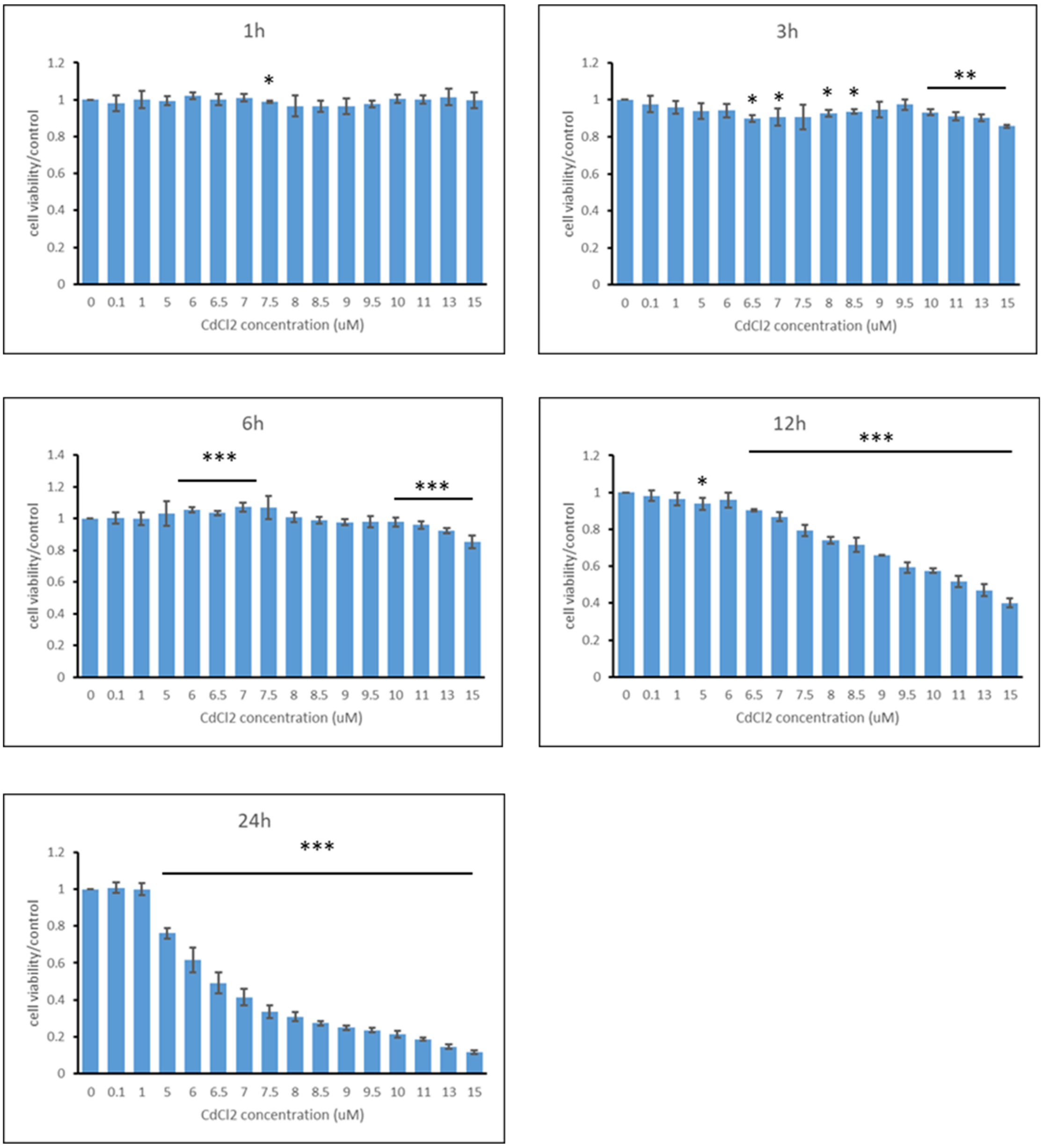

Figure S2: Morphological changes of TM4 after incubation with $CdCl_2$

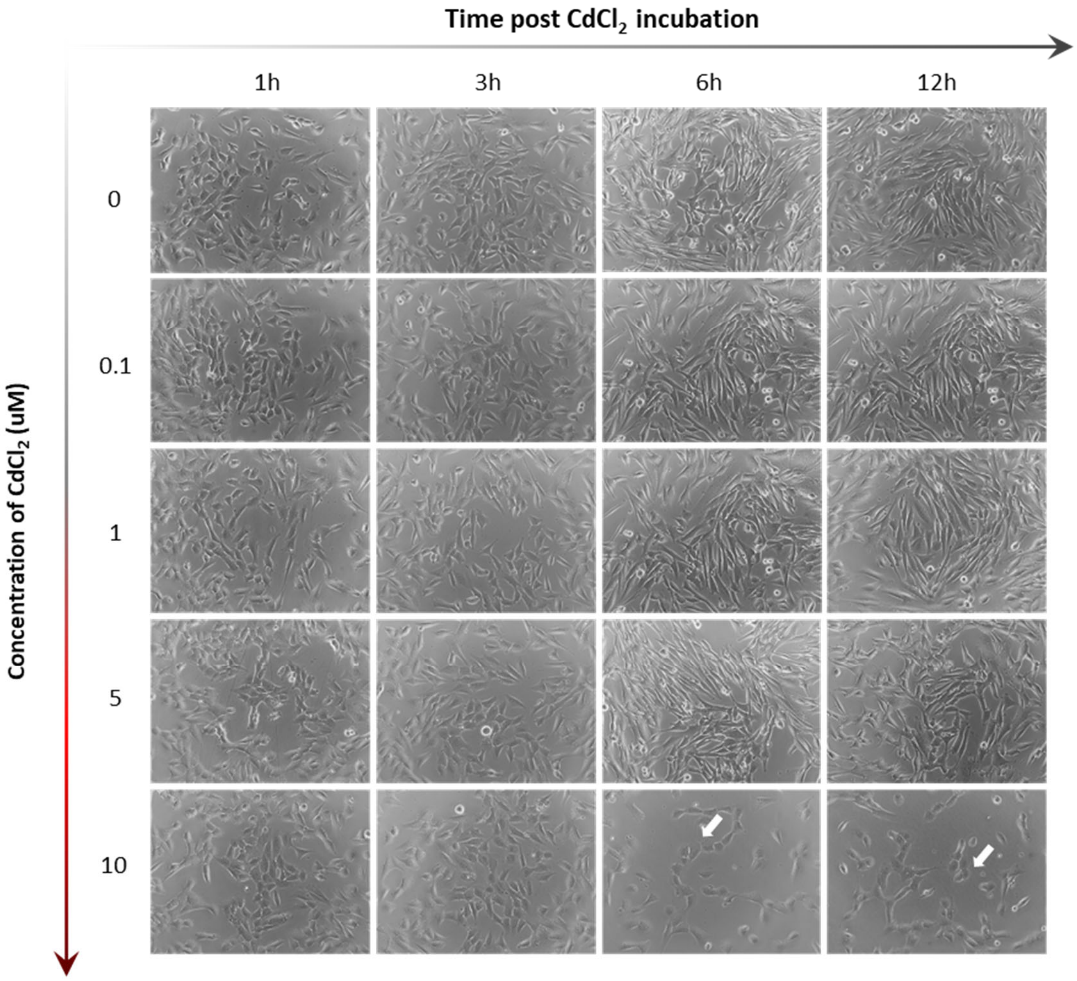

Figure S3: Transcriptional change of SOD1, Mff, and Tram

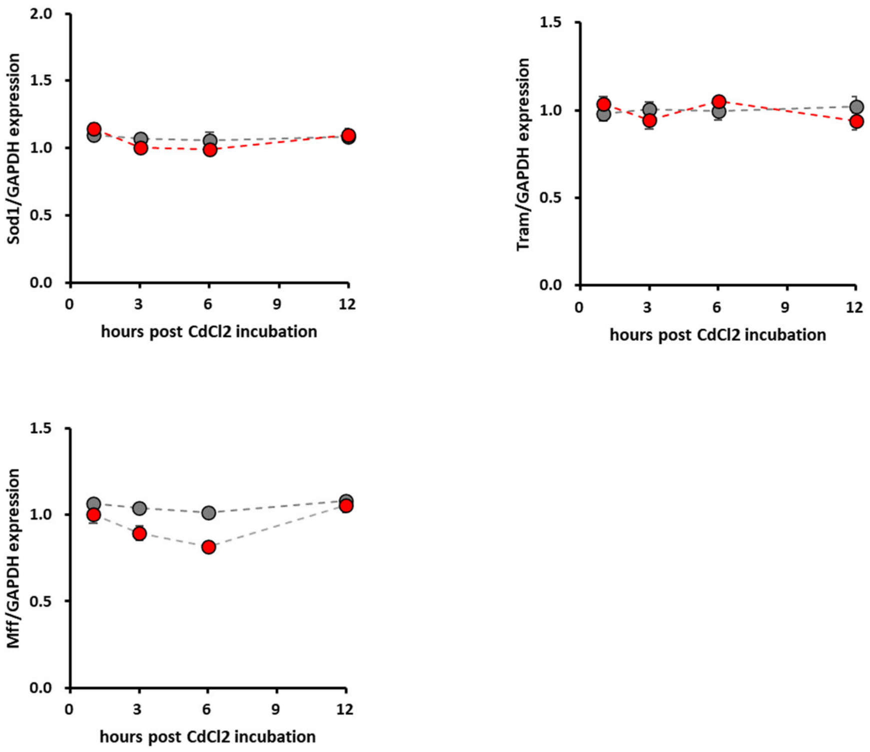

Atg5

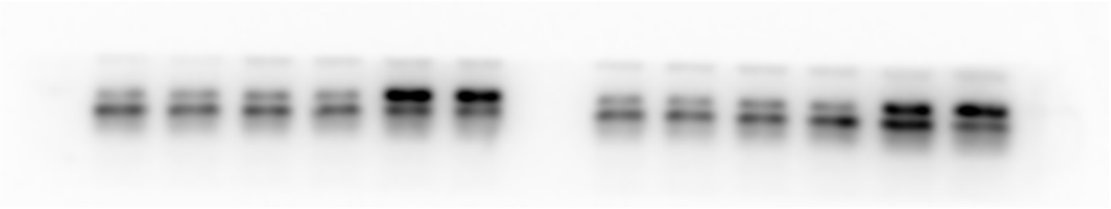

GAPDH1

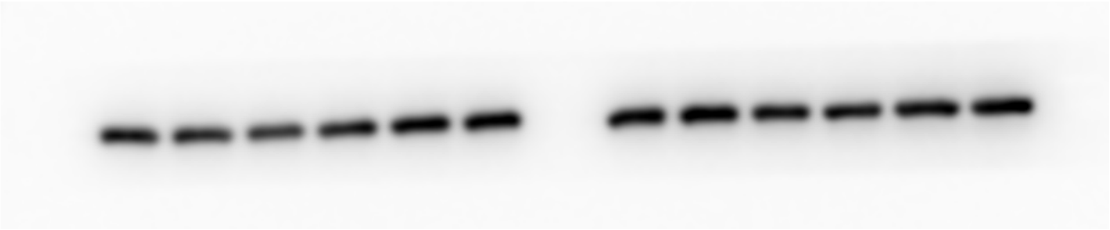

GAPDH2

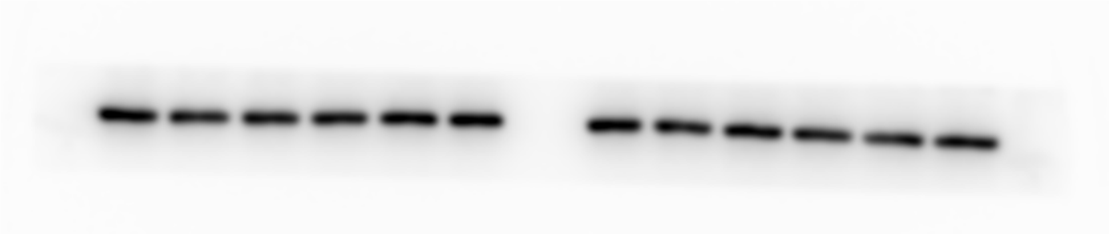

HO1

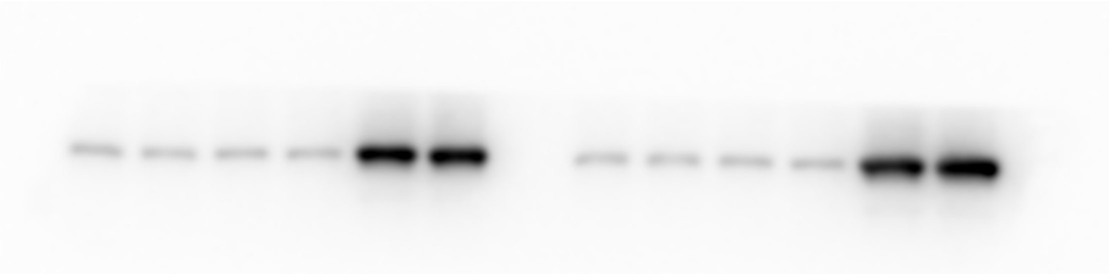

P62

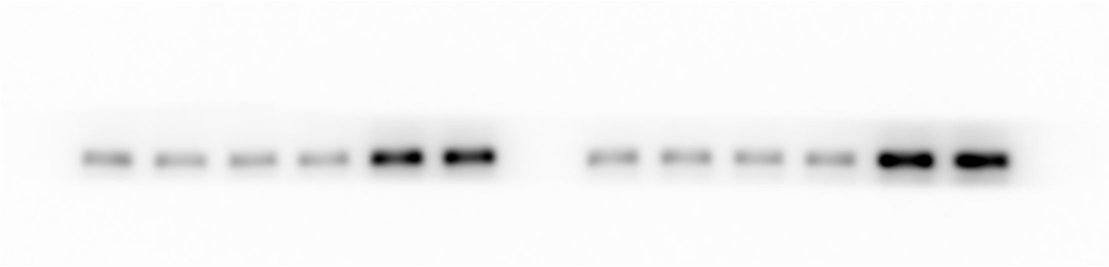

SOD

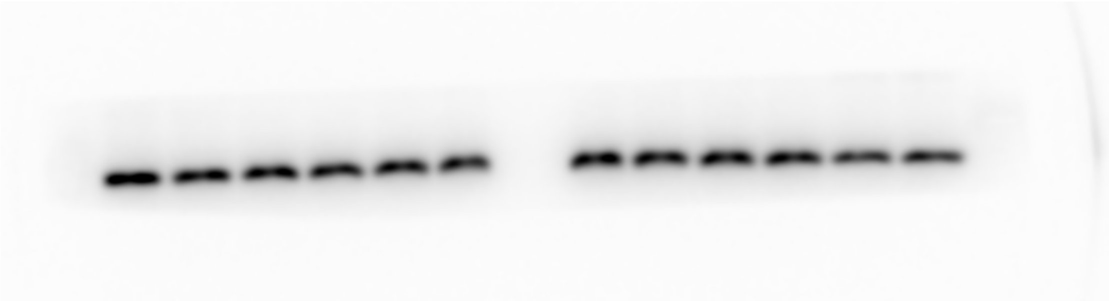

LC3

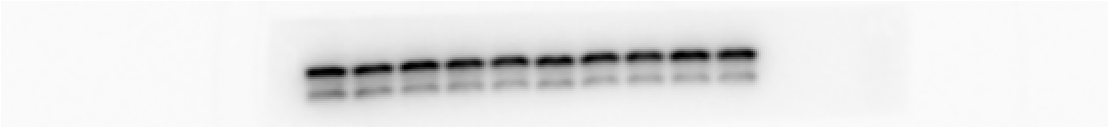

CHOP

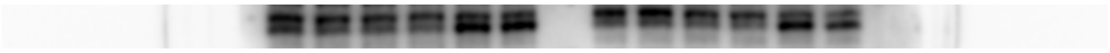

Hrf1

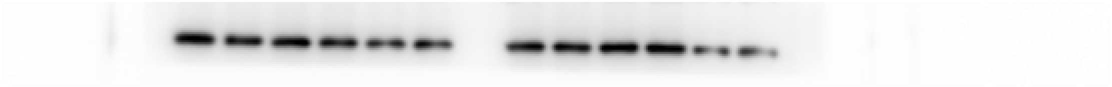

Mfn1

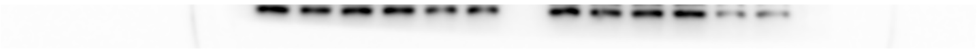

Supplement: Supplementary file 1 [file toxics-11-00167-s001.zip › toxics-2194327-supplementary.pdf]
